# Comparative study on the clinical effect of preparing neobladder with different lengths of ileum

**DOI:** 10.3389/fonc.2022.972676

**Published:** 2022-10-17

**Authors:** Bin Zheng, Zhenghong Liu, Heng Wang, Jinxue Wang, Pu Zhang, Dahong Zhang

**Affiliations:** Urology and Nephrology Center, Department of Urology, Zhejiang Provincial People’s Hospital, Affiliated People’s Hospital, Hangzhou Medical College, Hangzhou, China

**Keywords:** bladder tumor, radical cystectomy, ileal neobladder surgery, bladder volume, bladder function

## Abstract

**Objective:**

To investigate the relationship between orthotopic U-shaped ileal neobladder volume and bladder function. To investigate the correlation between the volume of the radical cystectomy and the U-shaped ileal neobladder *in situ* and the function of the bladder.

**Methods:**

The clinical data of patients undergoing in orthotopic U-shaped neobladder in our hospital were retrospectively analyzed. They were divided into two groups according to the length of the retained ileum. Group 1: The length of the ileum was 25-35cm (including 35cm), and the second group: the length of the ileum was 35-45cm. The basic information, cushion usage, urodynamic examination and complications of the two groups were obtained.

**Results:**

A total of 88 patients were included in the study, including 33 in the first group and 55 in the second group. There was no statistical difference in general data, lymph node collection, lymph node positive rate, positive margin rate, postoperative pathological stage, pathological grade, pathological type, intraoperative blood loss, blood transfusion, postoperative hospital stay, and complications between the two groups of patients. significance. Although the usage of urine pads in group 1 was more than that in group 2 in the short term after operation (P<0.05), it started from the third year after operation. Patients in group 1 used less cushion than group 2 (P<0.05). Urodynamic examination was performed on the patients, and the bladder function of group 1 maintained satisfactory time longer than that of group 2. The total number of deaths in the two groups was 12 and 23, respectively. The 5-year overall survival (OS) rate of group 1 was 53.60%, and the 5-year overall survival rate of group 2 was 52.9%.

**Conclusions:**

A new bladder formed by cutting the ileum with a length of 25-35 cm (including 35cm) has a longer time to maintain good bladder function than cutting the ileum with a length of 35-45 cm to produce a new bladder.

## Introduction

Currently, urinary reconstruction is divided into two categories: incontinence diversions, including ileal conduit (Bricker) diversion, and continental diversions, such as skin reservoirs and orthotopic neobladder connected to the urethra (1). In orthotopic neobladder surgery is the closest to a normal bladder in terms of anatomy and function, does not require an abdominal wall stoma, maintains a good personal image, and improves the quality of life. It has been popularized and applied in recent years (2, 3). Urinary control after neobladder surgery is mainly affected by the anatomical preservation of intraoperative neurovascular and sphincter. We retrospectively analyzed the clinical data of 88 patients with locally advanced bladder cancer in our hospital from May 2014 to May 2019. The patients were divided into two groups according to the length of the ileum intercepted during the operation, thereby affecting the size of the bladder capacity. The length of the intercepted ileum in one group was 25-35cm, and the length of the intercepted ileum in the second group was 35-45cm. To explore the correlation between different bladder capacity and bladder function.

## Materials and methods

This study reviewed 88 cases of locally advanced bladder cancer (cT2-3) admitted in our hospital from May 2014 to May 2019. The inclusion criteria included patients with better urinary control through preoperative urodynamic examination. Patients complete a follow-up for 2-7 years after surgery. The patients were divided into two parallel observation groups according to the length of the ileum intercepted during the operation. Observation group one: 33 cases, 30 males and 3 females; age from 48 to 77 years old with an average age of 60 years; Observation group 2: A total of 55 cases, 50 males and 5 females; aged 46-82 years old, with an average age of 64 years old; the two groups of patients have different ileum interception lengths. Observation group one: the length of the ileum cut was 25-35cm; the observation group two: the length of the cut ileum was 35-45cm.

The operations were performed by the same team of experienced doctors. Both groups of patients were under general anesthesia, and the patients were in a supine position. After entering the pelvic cavity through an abdominal incision, they underwent lymph node dissection and cystectomy. Separate a section of ileum from the ileocecal area, and perform a U-fold folding with the extremities facing the patient’s head. A mechanical stapler is used for lateral intestinal-intestinal anastomosis to establish intestinal continuity. The separated intestinal segment is folded in a U-shape, and the jaws of the mechanical stapler are placed into the intestinal segment opening for cutting and anastomosis. In order to complete the pouch to the tube, an opening is made at the lowest point of the U-shaped ileum, and the jaws of the mechanical stapler pass through the opening to complete the U-bag.

We use the cutting closure device to make the U-shaped neobladder of the ileum. This method reduces the length of the intestine required and reduces the tension between the urethra and the anastomosis of the neobladder. The neurovascular bundle is preserved during the operation. After the operation, a new bladder is flushed through a catheter to dilute the ileal mucus and prevent the catheter from being blocked. Postoperative angiography was performed to evaluate reflux and leakage.

We collected and evaluated the basic data of the two groups of patients, the clinical stage of the tumor, the volume of the new bladder, the residual urine volume, the maximum urine flow rate, the recovery of postoperative urinary control, and postoperative complications. The standard for good urinary control after surgery: the number of urine pads used is less than or equal to 1 piece (4), and the number of urine pads used for poor urinary control is> 1 piece. Detect maximum bladder volume, residual urine volume, maximum urine flow rate, etc. according to the technical report standards of the International Association of Urological Control. The bladder function of the two groups was compared through regular outpatient check-ups and telephone follow-up.

Postoperative follow-up was conducted in the form of telephone contact and outpatient follow-up, with an interval of 3 months in the first year, 6 months in the second year, and annual follow-up. The bladder function training after the operation is carried out about 1 week after the operation, and the urinary catheter is clamped and opened regularly. The time starts from every half hour and gradually opens once every 2 to 3 hours. The urine output varies from low to high, until about 250 mL of urine is excreted from the catheter each time. The urination habit training is carried out after the catheter is removed. According to the patient’s living habits and activity requirements, a urination plan is formulated. Generally, the patient is instructed to urinate 6 to 8 times during the day and 2 to 3 times at night. Voiding pattern training: They are taught to empty the neobladder by increasing intra-abdominal pressure and relaxing the pelvic floor. Urinary continence training: by repeatedly contracting and relaxing the pelvic floor muscles, to restore urinary continence as soon as possible and eliminate urinary incontinence.

The SPSS 25.0 software was used to statistically process the data, and the Kruskal-Wallis, T test and X2 test were used to analyze the data. The difference was statistically significant with P<0.05.

## Results

We evaluated 88 patients who underwent surgery. The median follow-up time of 33 patients in observation group 1 was 44 months (27-65 months), and the median follow-up time of 55 patients in observation group 2 was 65 months (42-82 months). There was no significant difference in the follow-up time between the two groups (P>0.05). The BMI of most patients was within the normal range, and the difference between the two groups was not statistically significant. There was no significant difference in the clinical staging of tumors between the two groups. There was also no significant difference in diabetes and postoperative pathological lymph node positive between the two groups (**Table S1**).

There was no significant difference in preoperative hematological indexes (including Cr, BUN, HP) between the two groups. The operation time of group 1 was slightly shorter than that of group 2, but the difference was not statistically significant. There was no significant difference in estimated intraoperative blood loss between the two groups (344.0 ± 159.0 *VS* 359 ± 163.1 P=0.629). The number of intraoperative or postoperative blood transfusions in group 2 was more than that in group 1, but there was no significant difference in blood transfusion between the two groups. The postoperative hospital stay was 16.0 ± 3.3 *VS* 17.8 ± 4.4 in the two groups respectively, and the difference was not statistically significant. There was no significant difference in early postoperative complications and late complications (**Table S2**)

The results showed that in our perioperative complications study, the electrolyte disturbance in group 1 was less than that in group 2 (P <0.05), and the incidence of hydronephrosis in group 2 was significantly higher than that in group 1. The time of exhaust and defecation in group 1 was significantly earlier than that in group 2, and there was no significant difference in other complications between the two groups. (**Table 1**).

**Table 1:** Perioperative and postoperative information of the two groups.

Variable	Group1	Group2	Pvalue
Perioperative complications			
Infection	7	12	0.947
Gastrointestinal tract related	3	8	0.677
Urinary fistula	2	2	0.629
Disturbance of electrolyte	9	27	0.044
Postoperative complications			
Infection	5	10	0.714
Chronic pyelonephritis	3	10	0.394
Kidney seeper	3	17	0.036
Bladder calculi	5	10	0.714
Gastrointestinal tract related	3	7	0.862
Time to flatus	35.5 ± 16.5	44.5 ± 17.2	0.013
Time to bowel	116.1 ± 21.3	125.7 ± 23.9	0.045

The usage of the changing pad reflects the bladder function of the patient. One year after the operation, the daily usage of urine pad in observation group 2 was less than that in observation group 1. At the same time, more than half of the patients have good urinary control ability during the day and only need to use the urine pad at night. With the prolongation of monitoring time, the use of urine pads in both groups improved significantly. By the second year after surgery, there was no statistically significant difference in the use of changing pads and the use of changing pads during the day and night between the two groups (P>0.05). Further follow-up, regardless of the comparison of the usage of the urine pad and the usage, the observation group 1 was significantly better than the observation group 2 (**Table 2**).

**Table 2:** Postoperative pathological results of bladder cancer patients in two groups.

Variable	Group 1	Group 2	P value
Surgical margins			0.629
Negative	31	53	
Positive	2	2	
Number of lymph nodes retrieved	18.0 ± 2.8	19.2 ± 3.5	0.099
Positive lymph node	2	2	0.629
Pathological stage			0.984
Tis	3	6	
T1	19	32	
T2	7	12	
T3	4	5	
Tumor grade			0.607
G1	6	15	
G2	17	24	
G3	10	16	
Pathological type			0.333
Transitional cell carcinoma	31	50	
Squamous cell carcinoma	2	3	
Adenocarcinoma	1	2	

The use of changing pads reflects the patient’s ability to control urine. At 1 year after surgery, the daily usage of changing pads in group 2 was less than that in observation group 1. At the same time, more than half of the patients had good urination control during the day, and only needed to use a urine pad at night. With the extension of follow-up time, the use of urine pads in both groups was significantly improved. By the 2nd year after operation, the patients in the two groups were better than the patients in the first group in terms of the number of urine pads used and the use of the white night pads. With the extension of follow-up time, the number of patients in group 1 gradually decreased, while the use of pads in group 2 increased gradually after the third year of follow-up (**Table 3**).

**Table 3:** The use of changing pads by patients.

Variable	Group 1	Group 2	P value
One year after surgery			
Pads per 24 h			0.032
0-1	17/33 (51.5)	40/54 (74.1)	
≥2	16/33 (48.5)	14/54 (25.9)	
Pad use			0.017
Day only	0/33 (0)	0 (0)	
Night only	17/33 (51.5)	41/54 (75.9)	
Day and night	16/33 (48.5)	13/54 (24.1)	
Two years after surgery			
Pads per 24 h			0.351
0-1	22/31 (71.0)	40/50 (80.0)	
≥2	9/31 (29.0)	10/50 (20.0)	
Pad use			0.559
Day only	0 (0)	0 (0)	
Night only	24/31 (77.4)	42/50 (84.0)	
Day and night	7/31 (22.6)	8/50 (16.0)	
Three years after surgery			
Pads per 24 h			0.032
0-1	20/24 (83.3)	26/44 (59.1)	
≥2	4/24 (16.7)	18/44 (40.9)	
Pad use			0.367
Day only	1/24 (4.2)	2/44 (4.5)	
Night only	17/24 (70.8)	27/44 (61.4)	
Day and night	4/24 (16.7)	14/44 (31.8)	
No	2/24 (8.3)	1/44 (2.3)	
Four years after surgery			
Pads per 24 h			0.040
0-1	12/14 (85.7)	16/33 (48.5)	
≥2	2/14 (14.3)	17/33 (34.8)	
Pad use			0.004
Day only	1/14 (7.1)	1/33 (3.0)	
Night only	8/14 (57.1)	13/33 (39.4)	
Day and night	2/14 (14.3)	19/33 (57.6)	
No	3/14 (21.4)	0/33 (0)	
Five years after surgery			
Pads per 24 h			0.021
0-1	6/7 (85.7)	8/21 (38.1)	
≥2	1/7 (15.3)	13/21 (61.9)	
Pad use			0.048
Day only	0 (0)	0 (0)	
Night only	5/7 (71.4)	6/21 (28.6)	
Day and night	1/7 (14.3)	15/21 (71.4)	
No	1/7 (14.3)	0	

The size and wetness of the pad are important indicators of urinary incontinence. In our study, the size of day and night pad use in group 1 was significantly smaller than that in group 2. It was found that there was no significant difference between group 1 and group 2 in the degree of pad wetting during the day (P =0.073). However, the pads were significantly wetter at night in group 2 than in group 1 (**Table 4**).

**Table 4:** Patterns of mucus leakage for two groups.

Variable	Group1	Group2	P value
Pad size(daytime)			<0.00
No use	6/7	6/21	
Small	0/7	1/21	
medium	1/7	8/21	
Large	0/7	6/21	
Pad size(nighttime)			0.042
No use	1/7	0/7	
Small	3/7	4/21	
medium	1/7	14/21	
Large	2/7	3/21	
Pad wetness(daytime)			0.073
No use	6/7	6/21	
Almost dry	0/7	1/21	
Slightly wet	1/7	3/21	
Wet	0/7	3/21	
Soaked	0/7	8/21	
Pad wetness(nighttime)			0.025
No use	1/7	0/7	
Almost dry	1/7	2/21	
Slightly wet	4/7	3/21	
Wet	1/7	10/21	
Soaked	0/7	6/21	

Within 1 year after the completion of radical bladder resection and orthotopic U-shaped neobladder, there was no significant difference in residual urine between the two groups (P>0.05). Subsequently, the bladder residual urine in observation group 1 was significantly less than that in observation group 2, and the difference in average residual urine volume between the two groups gradually increased. The residual urine volume of observation group 2 first showed a downward trend, and gradually increased in the 4th year after surgery. The maximum urine flow rate can reflect bladder function. The average maximum urine flow rate of observation group 1 after operation showed an upward trend. By the fifth year after operation, the average maximum urine flow rate was 19.0 ± 2.3 mL/s. However, the peak of the maximum urine flow rate in observation group 2 was in the 4th year after surgery. Too large or too small a new bladder will affect the function of the bladder. The longer it stays within a certain range, the more beneficial it will be to the patient’s postoperative urine control. Due to the different length of the intercepted ileum between the two groups of patients, the maximum bladder volume after the operation of the two groups has always been different (**Table 5**).

**Table 5:** Patient’s urodynamic parameters.

Variable	Group 1	Group 1	P value
One month after surgery	n=33	n=55	
Residual urine	66.4 ± 16.4	75.8 ± 19.4	0.296
Maximum flow rate (mL/sec)	8.8 ± 2.1	9.2 ± 1.8	0.169
Maximum reservoir capacity (mL)	232.6 ± 27.0	312.4 ± 24.4	0.000
Six months after surgery	n=33	n=55	
Residual urine	68.5 ± 12.3	67.5 ± 17.5	0.581
Maximum flow rate (mL/sec)	10.3 ± 2.4	12.4 ± 2.1	0.542
Maximum reservoir capacity (mL)	305.0 ± 28.3	387.1 ± 27.1	0.000
One year after surgery	n=33	n=54	
Residual urine	55.0 ± 14.5	58.3 ± 25.2	0.826
Maximum flow rate (mL/sec)	12.9 ± 2.8	13.1 ± 1.2	0.740
Maximum reservoir capacity (mL)	361.0 ± 33.2	440.1 ± 30.1	0.000
Two years after surgery	n=31	n=50	
Residual urine	42.8 ± 17.1	54.1 ± 44.3	0.038
Maximum flow rate (mL/sec)	15.8 ± 3.6	15.2 ± 3.5	0.310
Maximum reservoir capacity (mL)	390.1 ± 31.1	471.1 ± 33.6	0.000
Three years after surgery	n=24	n=44	
Residual urine	36.3 ± 20.7	51.3 ± 29.6	0.036
Maximum flow rate (mL/sec)	17.5 ± 3.8	15.9 ± 3.7	0.040
Maximum reservoir capacity (mL)	413.6 ± 34.8	509.1 ± 35.1	0.000
Four years after surgery	n=14	n=33	
Residual urine	29.8 ± 21.7	55.4 ± 37.6	0.018
Maximum flow rate (mL/sec)	18.6 ± 2.8	17.0 ± 3.1	0.023
Maximum reservoir capacity (mL)	443.9 ± 28.8	543.2 ± 36.5	0.000
Five years after surgery	n=7	n=21	
Residual urine	23.1 ± 15.3	64.0 ± 58.0	0.036
Maximum flow rate (mL/sec)	19.0 ± 2.3	14.2 ± 3.5	0.001
Maximum reservoir capacity (mL)	456.0 ± 53.6	581.2 ± 41.3	0.037

**Figure 1:**
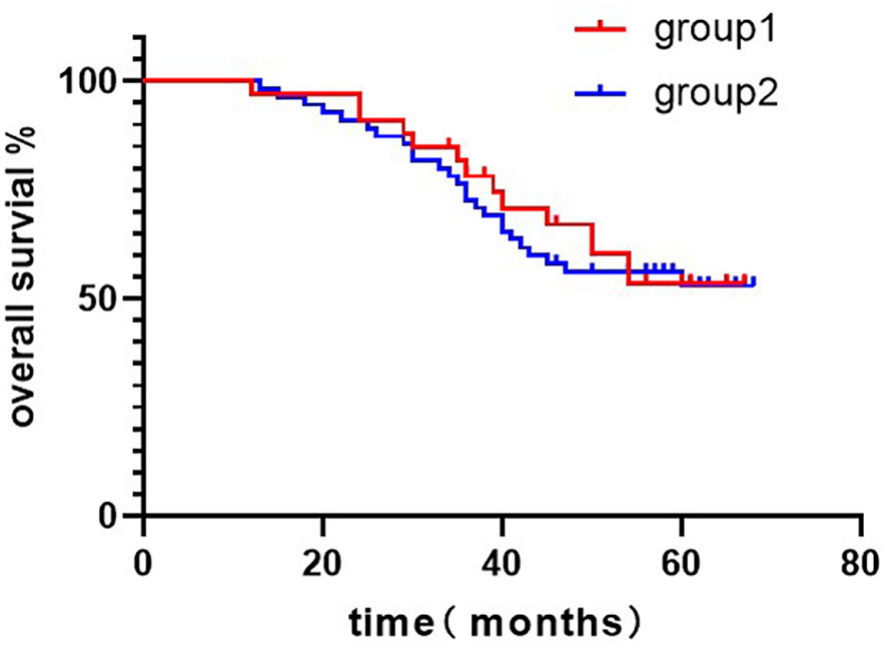
Kaplan–Meier curves of overall survival comparison in patients treated with group 1 versus group 2. 5-years-OS = (group 1: 53.60%, group 2: 52.80%; P = 0.657; HR = 0.855; 95CI = 0.429 to 1.705). Log-rank test: P=0.655.

The follow-up time of the patients in the two groups was 43.8 ± 13.0 and 47.5 ± 15.7 months, respectively, and the overall deaths were 12 and 23, respectively. The 5-year overall survival (OS)in group 1 was 53.60%, and the 5-year OS in group 2 was 52.90% (**Figure 1**). There was no significant difference in OS between the two groups.(P = 0.657; HR = 0.855; 95CI = 0.429 to 1.705).

## Discussion

Among urinary system tumors, the incidence or mortality of bladder cancer is extremely high. Radical cystectomy (RC) + urinary diversion (UD) is the gold standard for the treatment of muscular invasive bladder cancer and high-risk non-muscular invasive bladder cancer (5). As the age of onset of bladder cancer tends to be younger and medical conditions improve, many patients still have strong social requirements at the time of onset. RC+traditional urinary diversion surgery requires an abdominal wall stoma and an external urine bag, which affects the quality of life of patients after surgery. With the rise of the concept of urinary system reconstruction, especially the development of “in orthotopic ileal neobladder” surgery, bladder orthotopic reconstruction as a controllable urinary diversion surgery is gradually being carried out in various medical centers. However, this procedure takes a long time and the surgical technique is difficult, leading to its slow development. For this reason, urologists are actively exploring bladder reconstruction techniques in order to build an “ideal” new bladder with a shorter operation time and smaller abdominal incisions. Experience has shown that non-absorbable titanium nails have been safely used in various urology laparotomy and laparoscopic removal and reconstruction operations, including bladder cuff resection in nephroureterectomy. Based on this, Abreu (4) and others reported their ileal neobladder operation, which has the characteristics of “simple, fast and effective”. Using a mechanical stapler can quickly establish a new ileal bladder and significantly reduce the operation time. Inspired by this research, we carried out laparoscopic use of mechanical stapler and closing device to establish ileal neobladder.

There are some technical difficulties in the operation of RARC combined with urinary diversion. Previous experience shows that RARC is feasible to combine with urinary diversion, and is superior to LRC and ORC in many aspects (including Urinary function) (6–8). The team has rich LRC/RARC operation experience. In our previous research, we found that both RARC and LRC are safe and effective (9).

Initially, we used a 35-45 cm ileum for neobladder preparation during laparoscopic in orthotopic U-shaped ileal neobladder surgery. During the postoperative follow-up of the patient, it was found that the bladder function did not meet our ideal expectations. After analysis, it was found that the new bladder capacity expanded too quickly with time, and the time to maintain good bladder function was too short. To this end, we actively explored a shorter ileum for neobladder preparation, and finally decided to use a 25-35cm length of ileum for neobladder preparation.

After orthotopic bladder replacement, the most noteworthy issues include urinary tract changes and urinary dysfunction. As urine fills the new bladder, it acts as a low-pressure reservoir. During urination, the pressure on the abdomen, renal pelvis, and bladder increase at the same time, and the use of Valsalva will promote the emptying of urine without reflux (10, 11). In addition, the unidirectional peristalsis of the ureter and proximal segment of the ileum acts as a dynamic anti-reflux system during the filling phase (12).

In this study, 88 patients who used bladder substitutes were followed up for at least 2 years. In the comparison of the rate of daytime urinary incontinence, the incontinence rate of observation group 1 was higher than that of observation group 2 in the first year after operation (36.4% *VS* 23.6%); by the second year after operation, the difference in the rate of day incontinence between the two groups decreased (24.2% *VS* 16.4%); from the third year after the operation, the day incontinence rate of observation group 1 was always lower than that of observation group 2 during the follow-up period: (3rd year after operation: 8.0% *VS* 9.1%; fourth year after operation: 5.9% *VS* 13.0%; the fifth year after surgery: 0% *VS* 34.3%);. The nocturnal incontinence rate of the two groups also showed the above trend, (the first year after surgery: 42.4% *VS* 27.3%; the second year after surgery: 27.3% *VS* 25.5%; the third year after surgery: 20.0% *VS* 23.6%; the first year after surgery: 20.0% *VS* 23.6%; Four years: 11.8% *VS* 34.8%; fifth year after surgery: 14.3% *VS* 37.1%). According to reports, the urinary control rate after Intracorporeal orthotopic neobladder is 80-100% during the day and 45-90% at night (13–15). This difference may be due to the inconsistent definition of urinary incontinence and the inconsistent initial bladder capacity.

Other data also show that the control of urination during the day is better than that at night (16). It may be related to the loss of local spinal cord reflex arc, decrease of striated muscle tone and nighttime diuresis. In our study, in the observation group, 100% daytime self-control and 88.2% night self-control were in the 5th and 4th year after surgery. In the observation group, 90.9% of the daytime self-control and 76.4% of the night self-control were in the 3rd year after the operation. Adequate capacity and high compliance of the new bladder may significantly improve the patient’s urinary control rate.

In the urodynamic study, the average residual urine volume after urination of the new bladder in observation group 1 gradually decreased with the extension of follow-up time, and was 22.4 ± 19.8 mL at the fifth year after surgery. The average residual urine volume of observation group 2 entered a plateau after a period of decrease, and even tended to rise. The maximum bladder capacity of the two groups of neobladder was gradually increasing, and by the fifth year of follow-up, they were 456.0 ± 53.6 and 581.2 ± 41.3 mL, respectively. The maximum urine flow rate of the two groups of patients gradually increased with the recovery of the external sphincter strength and other factors, and the maximum values were 19.0 ± 2.3 and 17.0 ± 3.1 mL/sec. From the analysis of urodynamic data, the bladder function of observation group 2 gradually recovered after the operation, but by the fourth to five years of follow-up, the increase of residual urine and the decrease of maximum urine flow rate indicated the decline of bladder function. The possible reason is that the new bladder is too large. Although the longest follow-up time in this study was 5 years, there were still some patients with short follow-up time. At the same time, the small number of enrolled patients is also the weakness of this study.

Radical cystectomy and orthotopic neobladder surgery still have obvious complications. And the reporting rate and types of complications vary greatly (17–20). Early complications include bleeding, pulmonary complications, gastrointestinal and urinary system infections. Late complications are mainly affected by urinary diversion, including pyelonephritis, renal atrophy, bladder stones, and anastomotic stenosis.

The incidence of neo-bladder bacterial colonization associated with residual urine is 40-80% (21–23). Then, as the disease progresses, it gradually develops into obvious pyelonephritis (24). In our study, 4 and 5 patients in the two groups had postoperative pyelonephritis (**Table 2**). In the case of excluding urinary tract obstruction, urinary bacterial culture and drug sensitivity can better control the infection.

Studies have shown that anastomotic stenosis is one of the serious complications of orthotopic neobladder, and its incidence is 2.49% (25, 26). Severe ureterintestinal anastomosis stenosis leads to moderate to severe hydronephrosis and affects renal function. Urethral neobladder anastomotic stenosis will affect the emptying of urine and can be treated by dilatation of the urethra or a second operation. Another complication that affects urine emptying is neurogenic bladder. Due to radical cystectomy to clean the pelvic floor lymph nodes, it may cause damage to the proximal urethra and pelvic floor nerves. Such patients need long-term pelvic floor muscle exercises. One of the main concerns of the stapled reservoirs used in this study is the formation of stone. In Fontana et al (27) and Porena et al (28) study, the median follow-up period of 20 months and stone incidence of 64 months were 6% and 16%. In our study, the incidence of bladder stones was higher than in previous studies. Although titanium staples are conducive to the formation of stones, we have also noticed in this study that some stone patients have urinary tract infections and urinary retention at the same time. Therefore, we believe that there are many reasons for the formation of stones, not only related to titanium staples, but urinary tract infection is also an important factor.

## Conclusion

In short, orthotopic ileal neobladder is technically feasible. Our results show that the 25-35cm ileum-made bladder takes longer to maintain good bladder function than the 35-45cm ileum-made bladder to maintain good bladder function. It provides a new choice for the selection of the truncated length of the orthotopic ileum bladder. Although the follow-up time of observation group 1 was shorter than that of observation group 2, and with the extension of time, the number of people included in observation group 2 gradually decreased, and there was a certain selection deviation in the data, which made the results of the study have certain limitations. However, this study has certain reference significance for obtaining the ileum length from the orthotopic ileal neobladder.

## Data availability statement

The raw data supporting the conclusions of this article will be made available by the authors, without undue reservation.

## Ethics statement

This study was reviewed and approved by Ethics Committee of Zhejiang Provincial People’s Hospital (2020QT261). The patients/participants provided their written informed consent to participate in this study.

## Author contributions

Study conception and design: BZ and ZL. Acquisition of data; JW and HW. Analysis and interpretation of data: BZ and ZL. Drafting of manuscript: BZ and ZL. Critical revision of manuscript: PZ and DZ. All authors contributed to the article and approved the submitted version.

## Funding

This study was funded by Medical Technology Plan of Zhejiang Province (grant number: 2021421701), Medical Technology Plan of Zhejiang Province (grant number: 2022497314), the Natural Science Foundation of Zhejiang Province (grant number: LQ21H160041), the Natural Science Foundation of Zhejiang Province (grant number: LBQ20H050001).

## Conflict of interest

The authors declare that the research was conducted in the absence of any commercial or financial relationships that could be construed as a potential conflict of interest.

## Publisher’s note

All claims expressed in this article are solely those of the authors and do not necessarily represent those of their affiliated organizations, or those of the publisher, the editors and the reviewers. Any product that may be evaluated in this article, or claim that may be made by its manufacturer, is not guaranteed or endorsed by the publisher.
