# Supplementary material for: Comparative study on the clinical effect of preparing neobladder with different lengths of ileum
Source: Front Oncol. 2022 Oct 17;12:972676. doi: 10.3389/fonc.2022.972676 (PMC9618620; doi:10.3389/fonc.2022.972676)
Supplement: Supplementary file 1 [file DataSheet_1.docx]

Table S1 Demographic and clinical characteristics of the two groups of patients

| Variable | Group 1（n=33） | | Group 2（n=55） | P value |
| --- | --- | --- | --- | --- |
| Median follow-up, mo | 45（12-60） | 50（13-60） | | 0.264 |
| Median age, yr | 61（48-77） | 64（46-82） | | 0.923 |
| Age n (%) |  |  | | 0.538 |
| ≤60 yr | 15（45.5） | 19（34.5） | |  |
| 60-70 yr | 14（42.4） | 25（45.5） | |  |
| ≥70 yr | 4（12.1） | 11（20.0） | |  |
| BMI, n (%) |  |  | | 0.055 |
| ≤25 kg/m2 | 23（69.7） | 33（60.0） | |  |
| 25–30 kg/m2 | 8（24.2） | 19（34.5） | |  |
| ≥30 kg/m2 | 2（6.1） | 3 （5.5） | |  |
| Diabetes, n (%) | 6（18.2） | 9 （16.4） | | 0.826 |
| Clinical staging of tumor |  |  | | 0. 0.816 |
| cTis | 3（9.1） | 6（10.9） | |  |
| cT1 | 8（24.2） | 18（32.7） | |  |
| cT2 | 14（42.4） | 20（36.4） | |  |
| cT3 | 8（24.2） | 11（20） | |  |

Table S2 Surgical outcomes and perioperative complications

| Variable | Group 1 | Group 2 | P value |
| --- | --- | --- | --- |
| Cr（X±Sμmol／L） | 100±33 | 102±61 | 0.485 |
| BUN（X±S mmol／L） | 4.2±0.5 | 4.0±0.4 | 0.309 |
| HP (X±S g/ml) | 134±22 | 130±19 | 0.228 |
| Median operating time（X±S h） | 5.1±0.7 | 5.4±0.6 | 0.042 |
| ntraoperative blood loss（X±S ml） | 344.0±159.0 | 359±163.1 | 0.629 |
| Intraoperative and postoperative blood transfusion |  |  | 0.862 |
| blood transfusion | 4 | 6 |  |
| No blood transfusion | 29 | 49 |  |
| postoperative hospital stay（X±S days） | 16.0±3.3 | 17.8±4.4 | 0.077 |
| Early postoperative complications |  |  | 0.730 |
| Minor, Clavien–Dindo grade I–II | 6 | 11 |  |
| Major, Clavien–Dindo grade III–IV | 1 | 2 |  |
| Late postoperative complications |  |  | 0.659 |
| Minor, Clavien–Dindo grade I–II | 10 | 24 |  |
| Major, Clavien–Dindo grade III–IV | 3 | 4 |  |
